# A phenanthrene derived PARP inhibitor is an extra-centrosomes de-clustering agent exclusively eradicating human cancer cells

**DOI:** 10.1186/1471-2407-11-412

**Published:** 2011-09-26

**Authors:** Asher Castiel, Leonid Visochek, Leonid Mittelman, Françoise Dantzer, Shai Izraeli, Malka Cohen-Armon

**Affiliations:** 1 The Neufeld Cardiac Research Institute, Department of Physiology and Pharmacology, Sackler Faculty of Medicine, Tel-Aviv University, 69978, Tel-Aviv, Israel; 2 Department of Human Molecular Genetics and Biochemistry, Sackler Faculty of Medicine, Tel-Aviv University, 69978, Tel-Aviv, Israel; 3 Imaging Unit, Sackler Faculty of Medicine, Tel-Aviv University, 69978, Tel-Aviv, Israel; 4 Cancer Research Center, Sheba Medical Center, Tel-Hashomer, 52621 Ramat-Gan, Israel; 5 Biotechnology and Cell Signaling, UMR7242, Ecole Superieure de Biotechnologie Strasbourg, F-67400, Illkrich-Graffenstaden, France

## Abstract

**Background:**

Cells of most human cancers have supernumerary centrosomes. To enable an accurate chromosome segregation and cell division, these cells developed a yet unresolved molecular mechanism, clustering their extra centrosomes at two poles, thereby mimicking mitosis in normal cells. Failure of this bipolar centrosome clustering causes multipolar spindle structures and aberrant chromosomes segregation that prevent normal cell division and lead to 'mitotic catastrophe cell death'.

**Methods:**

We used cell biology and biochemical methods, including flow cytometry, immunocytochemistry and live confocal imaging.

**Results:**

We identified a phenanthrene derived PARP inhibitor, known for its activity in neuroprotection under stress conditions, which exclusively eradicated multi-centrosomal human cancer cells (mammary, colon, lung, pancreas, ovarian) while acting as extra-centrosomes de-clustering agent in mitosis. Normal human proliferating cells (endothelial, epithelial and mesenchymal cells) were not impaired. Despite acting as PARP inhibitor, the cytotoxic activity of this molecule in cancer cells was not attributed to PARP inhibition alone.

**Conclusion:**

We identified a water soluble phenanthridine that exclusively targets the unique dependence of most human cancer cells on their supernumerary centrosomes bi-polar clustering for their survival. This paves the way for a new selective cancer-targeting therapy, efficient in a wide range of human cancers.

## Background

We have recently reported the selective eradication of human triple negative mammary cancer cells MDA-231 by phenanthrene derivatives (also acting as potent inhibitors of polyADP-ribose polymerases) [1]. These compounds included the phenanthridines PJ-34 and Phen and the isoquiniline Tiq-A. They were originally designed to protect neuronal cells in the central nervous system from cell death evoked by high activity of PARP-1 in response to DNA damage caused by brain injury, stroke or inflammation [2,3]. We found that these molecules cause G2/M transition arrest in the cell cycle of both mammary cancer cells MDA-231 and normal epithelial cells MCF-10. However, while G2/M arrest was permanent in the cancer cells, and was accompanied by their massive cell death, normal mammary epithelial cells overcame the cell cycle arrest and continued to proliferate normally in the presence of these phenanthrene derivatives [1]. The most potent compound was PJ-34, which also efficiently prevented the development of MDA-231 xenotransplants in nude mice without inducing detectable toxic effects in the animals [1].

The current results outline a mechanism that apparently underlies the exclusive cytotoxicity of PJ-34 in these human mammary cancer cells. We found that this molecule acts as a centrosomes de-clustering agent in cells with supernumerary centrosomes, which are most abundant in these mammary cancer cells and in most human cancers [4,5].

Bipolar centrosomes assembly during metaphase is crucial for bipolar spindle formation and accurate chromosomes segregation in cells undergoing mitosis [6,7]. To accomplish these tasks, cells dividing with more than two centrosomes have developed a yet unresolved molecular mechanism, clustering their extra-centrosomes at two poles [4,5,8]. Failure of this bipolar centrosome assembly causes multipolar spindle structures and aberrant chromosomes segregation that prevent normal cell division [5]. This may lead either to 'mitotic slippage' where cells 'slip' out of mitosis to re-enter G1 without satisfying the spindle assembly checkpoint (SAC), or to induction of 'mitotic catastrophe cell death' [5,9,10] or 'anaphase catastrophy' [10]. Although cell death induced by failure to accomplish mitosis has been well documented, the underlying molecular mechanisms are still poorly understood [5,10]

Extra centrosomes are most abundant in cells of human solid cancers and in some of the human hematological malignancies [4,5], whereas in normal somatic cells the number of centrosomes is restricted, via several control mechanisms, to two centrosomes per cell [8,9,11]. This difference between normal and cancer human cells raised the idea of using compounds that interfere with the bipolar clustering of extra centrosomes to achieve selective eradication of cancer cells without harming normal tissues [5,12].

Our findings confirm this hypothesis by identifying a molecule, which prevents extra centrosomes clustering in mitosis and exclusively eradicates human cancer cells with supernumerary centrosomes without impairing normal proliferating cells.

## Methods

### Cells and cell cultures

Human cancer cell lines included mammary triple negative (MDA-231) cells, lung (H1299), colon (DLD-1), ovarian (HeyA8), and pancreatic (Panc-1) cells. These cell-lines and the human epithelial cell line MCF-10A were supplied by ATCC, (Manassas, VA, USA). Human umbilical vein endothelial cells (Huvec) were supplied by PromoCell (Heidelberg, Germany), and human primary adipose-derived mesenchymal stroma cells were prepared from human thymus and kindly donated by Prof Jonathan Leor (Sheba Medical Center, Israel). The human cancer cell lines were chosen for our experiments as representatives of human cancer cells with high occurrence of extra-centrosomes.

Cancer cell lines were cultured in 6-well multi-dish plates (Nunc; Roskilde, Denmark) and maintained in a medium containing Dulbecco's Modified Eagle Medium (DMEM) containing 10% horse serum, 1% L-glutamine, and 1% Pen-Strep-Amphotericin B (Gibco, UK). Epithelial MCF-10A cells were cultured in DMEM/F12 (Gibco, UK) containing 6% fetal bovine serum (FBS), 0.02% epidermal growth factor (100 μg/ml; CytoLab, Rehovot, Israel), 2.8% hydrocortisone (50 μM; Sigma-Aldrich), 0.1% insulin (10 mg/ml; Sigma-Aldrich), and 1% Pen/Strep (Gibco, UK). Human endothelial cells, Huvec were cultured in endothelial cell growth medium (cat. # C22011; Promocell, Heidelberg, Germany) and used within 3 passages. Human primary mesenchymal stroma cells were prepared from thymus and cultured in DMEM/F12 (Rhenium) containing 15% fetal bovine serum (FBS). These cells were used within 5 passages.

### Treatments

**Figure 1:**
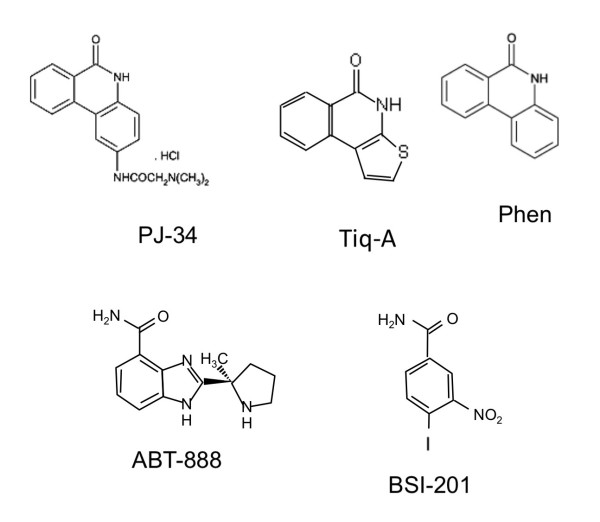
**The chemical structure of the compounds tested in both cancer and benign human cells**. The phenanthridines, PJ-34 and Phen, the isoquiniline Tiq-A, and the non-phenanthrene derivatives, ABT888 and BSI-201. These compounds act as potent PARP inhibitors.

Cells were treated with phenanthrene-derived compounds acting as potent PARP inhibitors: PJ-34 (N-(6-oxo-5,6-dihydro-phenanthridin-2-yl)-N,N-dimethyl-acetamide), Tiq-A, (4H-thieno[2,3-c]isoquinolin-5-one), and Phen, (6(5H)-phenanthridinone). PJ-34 and Phen were purchased from Alexis Biochemicals (Enzo Life Sciences International, Inc. PA, USA), Tiq-A was purchased from Sigma-Aldrich. In addition, cells were treated with two potent PARP inhibitors that are not phenanthrene derivatives, ABT-888 (2-[(R)-2-methylpyrrolidin-2-yl]-1H-benzimidazole-4-carboxamide) (Abbott Labs, Illinois, U.S.A) and BSI-201 (4-iodo-3-nitrobenzamide) (BiPar, San Francisco, CA, USA) (Figure 1).

**Flow cytometry **was performed with Beckton Dickenson machine and the FlowJo software (Tree Star, Ashland, OR). It was used to monitor changes in the ploidy levels of malignant and normal cells. At the indicated times, the cells were collected, permeabilized (75% ethanol in double-distilled water), and stained with propidium iodide. Cell eradication and the kinetics of S-phase entry and G2/M transition were monitored by measuring the percentages of cells at the different phases. Cell counts at the selected time periods during 120 hours incubation indicated cell-cycle changes resulting from treatment with the tested compounds. Untreated cells were used as controls for each cell type at the selected time periods.

### Confocal microscope imaging and immunocytochemistry

For immuno-cytochemistry, cells were grown on glass coverslips in the appropriate growth medium. After treatment, the cells were fixed with 4% paraformaldehyde (PFA; 20 minutes) and permeabilized with 0.2% Triton-X100 before blocking with 10% goat serum in PBS (details were described before [13]). The cells were immunolabeled for α and γ- tubulin. Spindles were labeled by antibodies directed against α-tubulin (T9026; Sigma) and detected by secondary fluorescent antibody Dylight (green) (Jackson ImmunoResearch Cat # 115-485-166). Antibodies directed against γ-tubulin (polyclonal, T5192; Sigma) detected by secondary fluorescent antibody CyTM3 (red) were used for immunolabeling of centrosomes. In some experiments centrosomes were double labeled for γ-tubulin (monoclonal, Abcam, MA, ab11316) and for centrin1 (polyclonal, Abcam, MA, ab11257) [14]. The percentage of multi-focal spindles was calculated in most cases out of a total number of 20 spindles that were detected in different randomly selected scanned samples of each of the tested cell types.

**For live confocal imaging of cells**, cells were transfected using liposomal reagent (Jet-PI, Polyplus, #101-10) with vectors encoding for γ-tubulin-GFP (for detection of centrosomes), α-tubulin-RFP (for detection of spindles and microtubule arrays) and for histone H2B-RED (for detection of chromosomes). Twenty-four hours after transfection the cells were exposed to PJ-34 (20 μM) for 18 hours, and then incubated in the presence of PJ-34 in live imaging chamber and scanned over-night (about 15 hours) by confocal microscope imaging. All the vectors were a gift of Prof. Michael Brandeis, Hebrew University, Jerusalem.

**Luminescent detection of ATP level in the cells **was used for quantification of cell viability. The Cell Viability Assay Kit (Abcam, MA) enabled a rapid quantification of viable cells in the cultures (the net result of growth arrest, cell death and cell proliferation). This method is based on the correlation between cell viability and ATP production, which signals the presence of metabolically active cells [15]. High percentage of cell death, as quantified by flow cytometry, was in correlation with decreased ATP levels in the tested cell cultures.

## Results

We examined several potent PARP inhibitors, including the phenanthrene derivatives PJ-34, Tiq-A and Phen and the non-phenanthrene derivatives ABT-888 and BSI-201 (Figure 1) in human cancer cell types with a high occurrence of extra-centrosomal cells (>50%).

In our previous experiments PJ-34 caused G2/M arrest and a massive cell death in MDA-231 mammary cancer cells without impairing normal human mammary epithelial MCF-10 cells [1]. Given that MDA-231 cells have high occurrence of extra-centrosomes, and the interference with bipolar clustering of supernumerary centrosomes causes G2/M arrest [5,16,17], we examined the possibility that PJ-34 affects extra-centrosomes clustering in mitosis.

**Figure 2:**
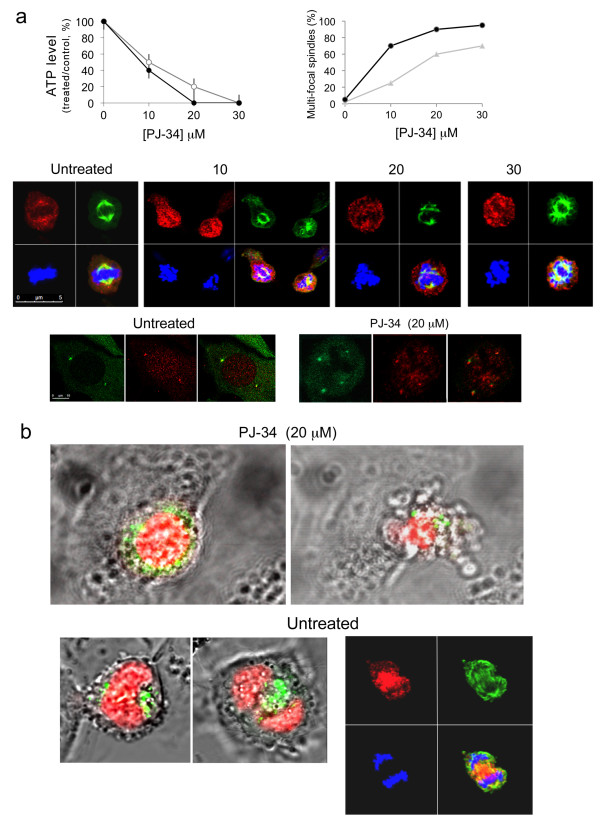
**De-clustering of supernumerary centrosomes in mitosis preceded PJ-34 induced cell death in human mammary cancer cells MDA-231**. **(a) **PJ-34 induced multi-polar spindles with scattered centrosomes and cell death in MDA-231 cells. **Top**: (*Left*) loss of cell viability after treatment with PJ-34 was quantified by luminescent detection of ATP levels in the treated cells (% ATP levels in cells treated with PJ-34 relative to ATP levels of untreated, control cells) (Methods). Cells were incubated with PJ-34 (applied once at 24 hours after seeding) for 72 hours (grey line) and 96 hours (black line). The mean values and standard deviations of 4 measurements in 3 different experiments are presented. *(Right*) The percentage of distorted spindles with scattered centrosomes in cells treated with PJ-34 was calculated out of 20 detected spindles by confocal scanning of randomly selected MDA-231 cells that were incubated with PJ-34 at the indicated concentrations and durations (48 hours; grey line) and (72 hours; black line). Both untreated and treated cells were permeabilized and immunolabeled for α- and γ-tubulin in 3 different experiments, as explained below. **Middle**: Confocal images presenting scattered centrosomes and aberrant chromosomes arrangement in multifocal spindles of randomly selected MDA-231 cells that were permeabilized and immunolabeled for α- and γ-tubulin for the detection of spindles and centrosomes (green and red fluorescent labeling, respectively), 18 hours after application of PJ-34 at the indicated concentrations. Chromosomes were labeled with DAPI reagent (blue) (Methods). **Bottom**: Double immunolabeling of centrosomes for γ-tubulin (green) and centrin1 (red) in randomly selected MDA-231 cells. Bipolar clustering of 4 centrosomes in an untreated cell (*Left*) and 4 un-clustered centrosomes in a cell treated with PJ-34 (20 μM) (*Right*) (Methods). **(b) **Live confocal imaging indicated the effect of PJ-34 on bi-polar centrosomes' clustering in randomly selected live MDA-231 cells. MDA-231 cells were transfected with vectors encoding for histone H2b-RED (red; for chromosomes labeling) and for γ-tubulin-GFP (green; for centrosomes labeling). Randomly selected cells were scanned overnight by confocal microscopy without or during treatment with PJ-34 (Methods). **Upper frame- **Un-clustered centrosomes (*Left*) during mitosis preceded cell death (*Right*) in a randomly selected live MDA-231 cell that was incubated with PJ-34 (20 μM) for 18 hours before scanning and during scanning (15 hours; imaging is included in Additional File 1). **Lower frame- **MDA-231 cells untreated with PJ-34. (*Left*) Live confocal images of randomly selected live MDA-231 cell with clustered centrosomes in anaphase. *(Right*) confocal image of randomly selected untreated fixed MDA-231 cell in anaphase. This cell was permeabilized and immunolabeled for α-tubuline (green; spindles) and for γ-tubuline (red; centrosomes). Chromosomes were labeled with DAPI reagent (blue).

Our experiments disclosed a remarkable activity of PJ-34 on disruption of the bipolar clustering of supernumerary centrosomes in mammary MDA-231 cancer cells, causing distorted multi-polar spindles as early as 6 hours after PJ-34 application, followed by massive cell death within 48-72 hours (Figures 2a, b and Additional File 1). PJ-34 was applied only once to the cells, 24 hours after seeding, at twice to 3-times higher concentration than that completely inhibiting the activity of PARP-1 [3]. The cytotoxic activity of PJ-34 was identified in randomly selected fixed or live MDA-231 cells, scanned by confocal microscopy (Figures 2a, b and Additional File 1; Methods). Also, PJ-34 did not seem to impair the structure or formation of centrosomes, as indicated by centrosomes double labeling with centrin1 and γ-tubuline (Figure 2a) and by confocal imaging of centrosomes in live MDA-231 cells in the interphase (Additional File 2).

These findings associated the remarkable cytotoxic activity of PJ-34 in MDA-231 cells with de-clustering of their extra-centrosomes in mitosis, causing multi-polar distorted spindles and aberrant chromosomes segregation that lead to 'mitotic catastrophe cell death' [5].

**Figure 3:**
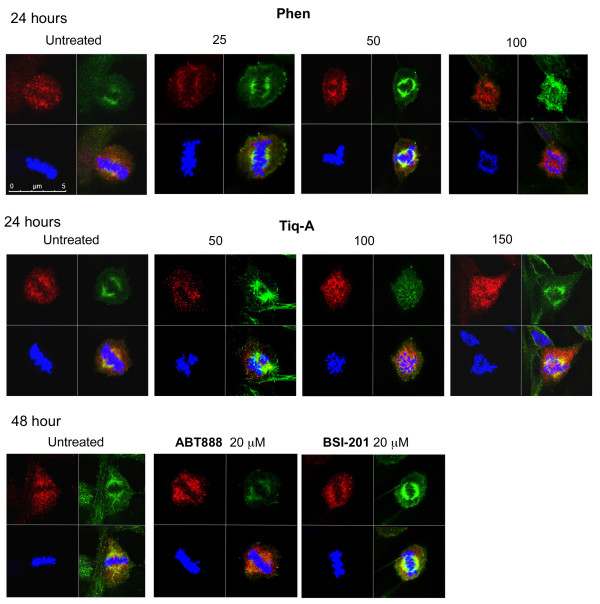
**The phenanthrene derivatives Phen and Tiq-A interfered with supernumerary centrosomes clustering in MDA-231 cells**. Confocal images present scattered centrosomes and aberrant arrangement of chromosomes in randomly selected fixed MDA-231 cells in mitosis. **Top**: These cells were permeabilized and immunolabeled for α- and γ-tubulin (labeling spindles (green) and centrosomes (red), respectively) 24 hours after application of Tiq-A or Phen at the indicated concentrations. **Bottom**: Bi-polar clustering of centrosomes in randomly selected fixed MDA-231 cells that were incubated for 48 hours with the non-phenanthrene derivatives ABT-888 and BSI-201, at the indicated concentrations, permeabilized and immunolabeled for α- and γ-tubulin. Chromosomes were labeled with DAPI reagent (blue).

Other phenanthrene derived molecules acting as PARP inhibitors, Phenanthridinon (Phen), and Tiq-A (Figure 1; [18,19]), similarly interfered with centrosomes clustering in MDA-231 cells. However, their effect was milder and accompanied by a lower percentage of cell death (Figure 3).

In contrast, non-phenanthrene derivatives acting as potent PARP inhibitors, BSI-201 and ABT888 [18,19], did not share this capability. Even when used at high concentrations (2-4 times higher than concentrations completely inhibiting the activity of PARP-1) they did not impair the bifocal clustering of extra centrosomes in these cells (Figure 3).

To further test the suggested contribution of PJ-34 to extra-centrosomes de-clustering, we examined its activity in other human cancer cell-lines with high occurrence of supernumerary centrosomes.

**Figure 4:**
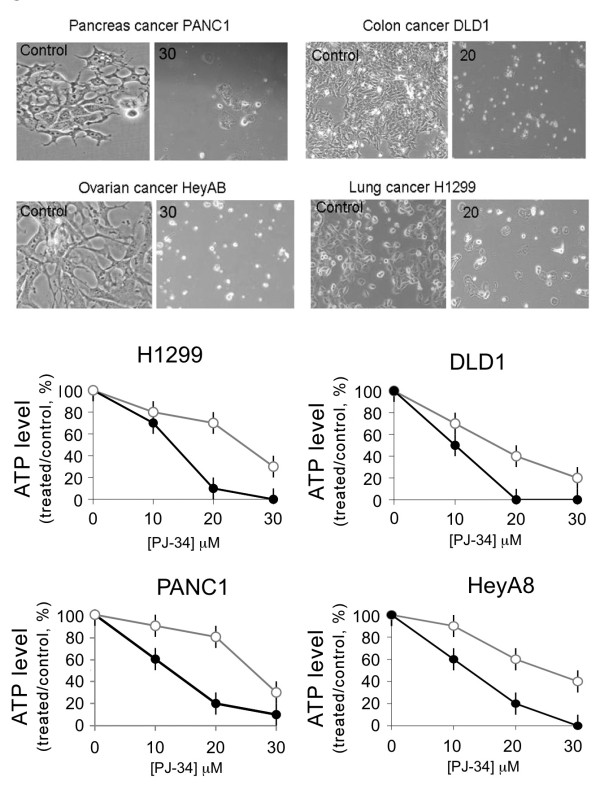
**Eradication of human cancer cells with high occurrence of extra-centrosomes by the phenanthridine PJ-34**. **Top**: Colon cancer cells DLD-1, lung cancer cells H1299, ovarian cancer cells HeyA8 and pancreatic cancer cells PANC-1 were examined under light microscope in randomly selected fields (0.02-0.03 mm^2 ^per field). The presented cells were either untreated (control) or incubated for 96 hours with PJ-34 applied once at 24 hours after seeding at the indicated concentrations. These are representative results of experiments that were repeated three times for each cell line. **Bottom**: Changes in cells' viability (total viable cells) were quantified by luminescent detection of ATP levels (% ATP level in cells treated with PJ-34 relative to ATP level measured in untreated, control cells) in each of the indicated cancer cell-lines. cells were incubated for 72 hours (grey line) and for 96 hours (black line) with PJ-34 applied once at 24 hours after seeding at the indicated concentrations (Methods). The mean values and standard deviations of 4 measurements in each cell-line in 3 different experiments are presented.

**Figure 5:**
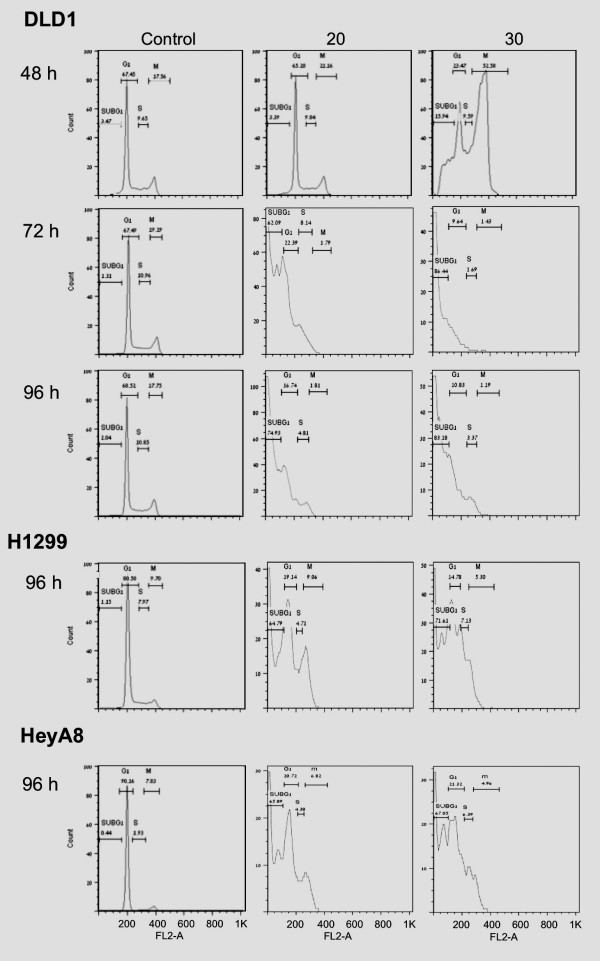
**Flow cytometry indicating G2/M arrest and cell death in the multi-centrosomal human cancer cells**. PJ-34 was applied 24 hours after seeding to human colon cancer DLD-1 cells, lung cancer H1299 cells and ovarian cancer HeyA8 cells at the indicated concentrations. Treated and control cells were analyzed 48, 72 and 96 hours after PJ-34 application (48 and 72 hours are presented only for DLD1). The effect of PJ-34 on cell eradication (sub-G1 population) and the kinetics of S-phase entry and G2/M transition were assayed by the percentage of cells at each of these phases. For each cell type, similar results were obtained in experiments repeated 3 times (Methods).

**Figure 6:**
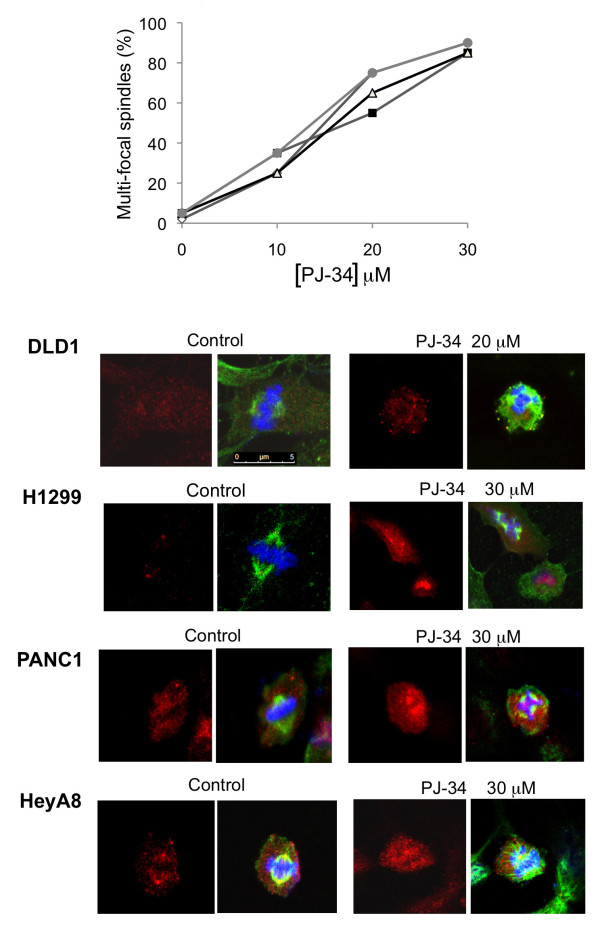
**The phenanthridine PJ-34 prevented bi-polar clustering of extra-centrosomes in the tested human cancer cells**. **Top**: PJ-34 interfered with the bifocal clustering of centrosomes in the indicated human cancer cells (DLD1, grey line, solid grey dots; H1299, grey line, empty diamonds; HeyA8, grey line, solid black squares; PANC1, black line, empty triangles) fixed 72 hours after application of PJ-34 at the indicated concentrations. Control and treated cells were permeabilized and immunolabeled for α- and γ-tubulin, for the detection of spindles (green) and centrosomes (red), respectively, as described below. The percentage of multi-focal spindles was calculated out of 20 spindles detected in randomly selected fixed cells by confocal microscopy in 3 different experiments. **Bottom**: Confocal images indicate scattered centrosomes and multi-focal spindles in randomly selected fixed cells in mitosis of the indicated cell-lines, 48 hours after application of PJ-34 at the indicated concentrations. Control (untreated) and treated cells were permeabilized and immunolabeled for α- and γ-tubulin (green and red, respectively). Chromosomes were labeled with DAPI reagent (blue).

PJ-34 similarly eradicated the tested cells, including lung (H1299), colon (DLD-1), ovarian (HeyA8), and pancreatic (Panc-1) cancer cells. PJ-34 (20-30 μM) caused G2/M arrest in these cells and a subsequent massive cell death, observed 48-96 hours after PJ-34 application (Figures 4 and 5). In accordance, PJ-34 prevented the bipolar clustering of supernumerary centrosomes in these cells, causing multipolar spindles and aberrant chromosomes alignment detected within 18 hours after PJ-34 application (Figure 6). For the tested five types of cancer cell-lines, there was a significant correlation between the fraction of cells with multi-polar spindles and the reduction in cell viability (as indicated by the reduction in ATP level) of the treated cells (Figures 2a, 4 and 6; r = 0.92; p < 0.0001). Thus, cell death could be attributed to de-clustering of their extra-centrosomes.

**Figure 7:**
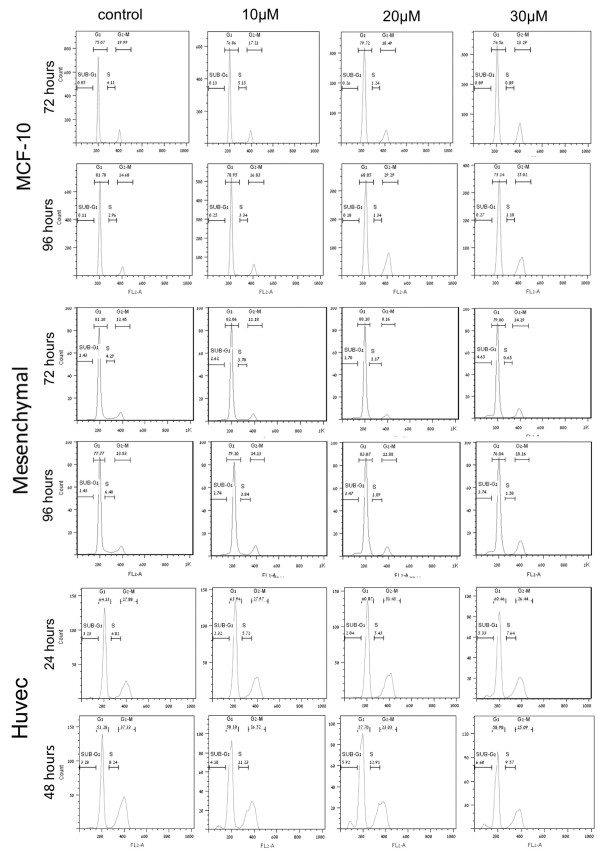
**The phenanthridine PJ-34 did not affect the cell-cycle of human normal proliferating cells**. Flow cytometry analysis shows that PJ-34 did not affect the cell cycle of human mammary epithelial cells MCF-10, adipose-derived mesenchymal stroma cells prepared from thymus, and human umbilical vein endothelial cells (Huvec). Cells were incubated for the indicated periods with the indicated concentrations of PJ-34 applied 24 hours after seeding. For each cell type, similar results were obtained in experiments repeated 3 times (Methods).

In contrast, normal human proliferating cells were resistant to the cytotoxic activity of PJ-34, Tiq-A and Phen in human cancer cells. Even at the high concentrations used and the long incubation periods, the most potent compound, PJ-34, did not interfere with the cell cycle of benign human proliferating cells. The tested cells included human mammary epithelial cells MCF-10, mesenchymal cells prepared from human thymus and human umbilical vein endothelial cells (Huvec) (Figure 7). As for Huvec, PJ-34 did not affect their cell cycle within 48 hours incubation (Figure 7). These cells did not survive longer incubation periods even without treatment (> 5% cell death was measured in untreated Huvec cells after 72 hours incubation).

**Figure 8:**
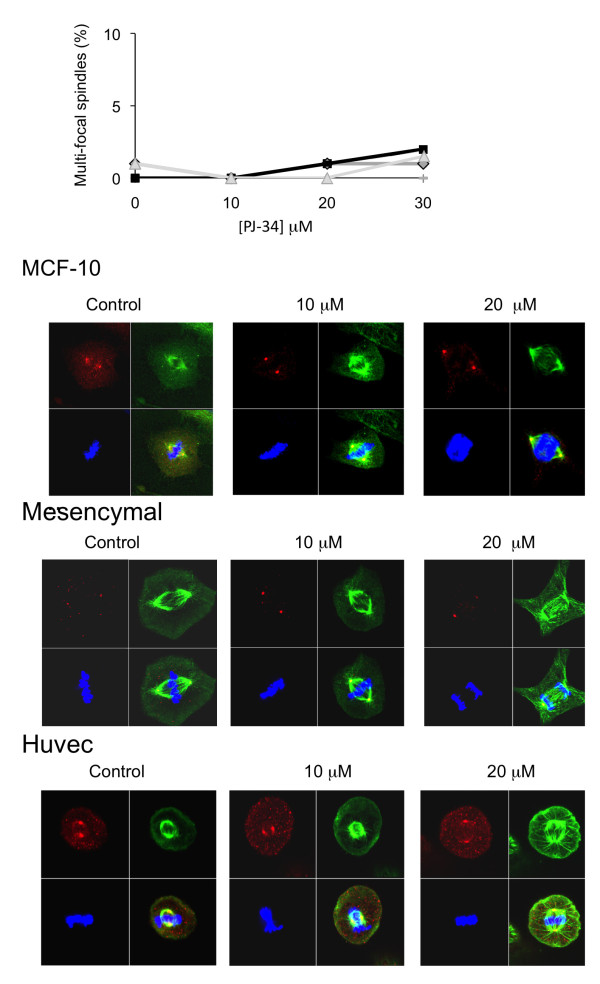
**Centrosomes assembly, bifocal spindles and chromosomes segregation were not impaired by PJ-34 in normal human proliferating cells**. Epithelial cells MCF-10, mesenchymal stroma cells and endothelial cells (Huvec) were scanned by confocal microscope after 72 hours incubation in the absence or presence of 20 μM PJ-34, applied 24 hours after seeding at the indicated concentrations. **Top**: Percentage of multi-focal spindles (approximately zero) in randomly selected fixed cells was calculated out of at least 20 detected spindles in 3 different experiments performed with each cell type (MCF-10, black line, solid black square; mesenchymal cells, grey line, solid grey diamond; Huvec, grey line, grey triangle). Control (untreated) and treated cells were permeabilized and immunolabeled for α- and γ-tubulin for the detection of spindles and centrosomes, respectively, as described below. **Bottom**: Confocal images of randomly selected cells in mitosis, untreated (control) cells, and cells that were incubated with PJ-34 for 72 hours at the indicated concentrations. Untreated cells and cells treated with PJ-34 were permeabilized and immunolabeled for α- and γ-tubulin (green and red, respectively) for detection of spindles and centrosomes, respectively. Chromosomes were labeled with DAPI reagent (blue).

In these three types of benign cells, PJ-34 did not interfere with the formation and assembly of centrosomes (two centrosomes at the poles of bifocal spindles), nor with chromosomes segregation. Bipolar assembly of centrosomes, bifocal spindle formation and chromosomes segregation were not impaired in the normal proliferating cells, even after long incubation with high concentrations of PJ-34 (Figure 8).

## Discussion

Our results suggest that the exclusive eradication of human cancer cells with extra centrosomes by the phenanthridine derivative PJ-34 is attributable to its extra-centrosomes de-clustering activity in mitosis (Figures 2, 4, 5, 6, Additional File 1 and Additional File 2).

According to our observations, PJ-34 did not impair centrosomes in both human normal and cancer cells, nor did it interfere with the formation of centrosomes in the interphase of multi-centrosomal cancer cells or impaired the bi-polar assembly of centrosomes in mitosis of normal proliferating cells with two centrosomes (Figures 2a, 8, Additional File 1 and Additional File 2). These features may underlie its outstanding capability to exclusively eradicate human cancer cells while keeping normal proliferating cells un-affected (Figures 5 and 7).

Theoretically, in cancer cells with high occurrence of extra-centrosomes (>50%) when mitosis is accompanied by eradication of multi-centrosomal cells, an exponential reduction of the fraction of bi-centrosomal cancer cells is expected. In this case, the higher their proliferation rate the more rapidly will these cancer cells be eradicated by extra-centrosomes de-clustering agents.

The indicated interference of the tested phenanthrene derivatives with the bipolar clustering of supernumerary centrosomes was not shared by non-phenanthrene derivatives acting as potent PARP inhibitors as well (Figure 3). This questions an exclusive role of PARP inhibition in extra centrosomes de-clustering. Nevertheless, these results could also reflect a possible, yet un-identified selective inhibition of PARP isoforms by phenanthridines.

Results obtained from genome-wide RNAi screen in multi-centrosomal cells, including embryonic *Drosophila *S2 cells and human malignant cells, identified genes required to suppress multipolar mitosis. Proteins involved in the organization and regulation of the cytoskeleton, including Kinesin HSET/Ncd (which is not required for polar organization in normal somatic cells) and the four chromosomal passenger complex (CPC) components, Aurora-B, INCENP, Survivin, and Borealin were identified among the main proteins required for bifocal clustering of extra-centrosomes in these cells [12,17]. Evidence for the necessity of tankyrase-I and a putative human PARP-16 homolog in centrosomes clustering has been also provided [17,20]. PolyADP-ribosylation of tankyrase-1 may contribute to spindle bipolarity by providing a static matrix, anchoring microtubule-associated motor proteins and spindle proteins [17,20]. A specific role for PARP-16 in mitosis has not been identified yet [17].

PARP proteins are present in centrosomes [21-24], and both Aurora-B and INCENP are targets for polyADP-ribosylation [22,24]. In addition, supernumerary centrosomes were found in PARP-1 deficient cells [25]. Thus, in view of the exclusive interference of PJ-34 with extra centrosomes de-clustering in mitosis, a possible synergism between PARP activity and other mechanisms underlying extra-centrosomes clustering should be further investigated.

The indicated cytotoxic activity of the phenanthrene derivatives in human cancer cells with high occurrence of extra-centrosomes links for the first time between two well known, but poorly understood phenomena; Some anti-tumor effects of natural phenanthridines were first described in the early fifties [26-28], and a higher cytotoxicity of some phenanthridines in cancer cells as compared to normal cells has been reported before [29,30], although the underlying mechanisms have never been understood. In addition, cell-cycle arrest resulting from failure of centrosomes to accomplish bipolar clustering in multi-centrosomal cells has been acknowledged for nearly a century [6,31,32]. Nevertheless, the causal relationship between multiple centrosomes and malignancy has only recently been clarified [32-35].

Mitotic-spindle microtubules are among the most effective targets for anti-cancer therapy [5,10,17,36-39]. Therefore efforts are invested in trying to find mechanisms exclusively targeting mitosis in cancer cells [40-42].

Unlike the exclusive interference of PJ-34 with extra centrosomes bi-polar clustering in mitosis (Figures 2, 3, 4, 5, 6, Additional File 1 and Additional File 2), the currently known small molecules interfering with mitosis in cancer cells target microtubule polymerization (vinca alkaloids, taxanes, colchicines, and also griseofulvin). These compounds therefore impair normal cells as well, and cause severe side effects [36-39].

The discovered potency of PJ-34 in preventing bipolar clustering of extra centrosomes in mitosis may pave the way for a new efficient therapy lacking intolerable side effects, which is based on compounds that exclusively target the unique dependence of many human cancer cells on their supernumerary centrosomes clustering for their survival.

Since many human cancer cells have high occurrence of multicentrosomal cells, the exclusive cytotoxic activity of PJ-34 in multi-centrosomal human cancer cells holds promise as a therapeutic tool for a selective chemotherapy in a wide range of human cancers, beyond the currently used selective therapeutic tools targeting specific genes/proteins that affect few cancer cell types [43-45].

## Conclusion

The identification of a molecule exclusively targeting the unique dependence of most human cancer cells on their supernumerary centrosomes bi-polar clustering for their survival, paves the way for a new highly selective cancer-targeting therapy, efficient in a wide range of human cancers.

## Competing interests

M. Cohen-Armon is the inventor of patent WO 2009/0477052, owned by the Tel-Aviv University. The co-authors declare that they have no competing interests. This study was not supported by any company or commercial fund.

## Authors' contributions

LV and AC performed the experiments SI and AC contributed new analytical tools. LM performed the confocal measurements, FD-proofreading of the manuscript. MC-A designed the experiments and wrote the manuscript.

All authors read and approved the final manuscript.

## Pre-publication history

The pre-publication history for this paper can be accessed here:

http://www.biomedcentral.com/1471-2407/11/412/prepub

## Supplementary Material

Additional file 1 **Live confocal imaging of MDA-231 cell in mitosis in the presence of the phenanthridine PJ-34**. Mitosis with scattered centrosomes ended by cell death in a randomly selected MDA-231 cell that was incubated for 18 hours with PJ-34 (20 μM) applied 24 hours after transfection by the indicated vectors, and then scanned over-night (approximately 15 hours) in the presence of PJ-34 by confocal microscope. Cells were transfected by vectors encoding for γ-tubulin-GFP (for labeling of centrosomes; green) and for histone H2B-RED (for labeling of chromosomes; red). Click here for file

Additional file 2 **Live confocal imaging of MDA-231 cell in interphase in the presence of the phenanthridine PJ-34**. Interaction between centrosomes in a randomly selected MDA-231 cell in interphase. This cell was incubated with PJ-34 (20 μM, 18 hours incubation) applied 24 hours after transfection by vectors encoding for γ-tubulin-GFP (green, for labeling of centrosomes) and for α-tubulin-RFP (red, for labeling of microtubule arrays in the cytoskeleton) and scanned overnight in the presence of PJ-34 (approximately 15 hours) by confocal microscope. Click here for file
